# Risk assessment of human-to-human transmission of severe fever with thrombocytopenia syndrome virus based on 10-year clustered analysis

**DOI:** 10.3389/fpubh.2024.1419425

**Published:** 2024-10-11

**Authors:** Nannan Zhang, Xiaodong Mu, Jingyu Liu, Tao Liu

**Affiliations:** Department of Infectious Disease Control, Yantai Center for Disease Control and Prevention, Yantai, Shandong Province, China

**Keywords:** severe fever with thrombocytopenia syndrome, SFTSV, human-to-human transmission, risk, cluster

## Abstract

**Background:**

Severe fever with thrombocytopenia syndrome (SFTS) is an acute infectious disease, which was first reported in 2009 in China. Previous studies have rarely quantitatively assessed the transmission and fatal risk of SFTS clusters.

**Methods:**

Epidemiological information regarding SFTS clusters in Yantai city of Shandong province during 2013-2022 was obtained from the National Public Health Emergency Event Surveillance System (PHEESS) for Disease Control and Prevention information system. The secondary attack rate (SAR) and relative risk (RR) were used to assess the risk of human-to-human transmission of SFTS.

**Results:**

A total of 20 SFTS clusters involving 51 laboratory-confirmed patients were reported between 2013 and 2022 in Yantai city, Shandong province. Most of the clusters occurred from May to October, and the patients were mainly distributed in four counties. Contact with blood or other fluids [RR = 14.06, 95% confidence interval (CI) = 3.29–70.65, *p* < 0.001] and using no personal protection equipment (PPE) [11.63% (10/86) vs. 2.22% (2/90), RR = 5.74, 95% CI = 1.17–55.44, *p* = 0.013] were significantly related with an increased risk of SFTS virus (SFTSV) transmission.

**Conclusion:**

Our study may provide direct guidance on health education and behavioral interventions for the accompanying relatives and personnel of SFTS patients, both during their hospital stay and upon returning home after discharge.

## 1 Introduction

Severe fever with thrombocytopenia syndrome (SFTS) is an acute infectious disease that seriously imposes substantial burden on public health. It was first reported in Hubei and Henan provinces in China from March to July 2009, and afterward, it rapidly spread to other provinces in the central, eastern, and northeastern regions (1). Later cases of SFTS were subsequently reported in countries of East Asia and South Asia (2, 3). In 2012, a novel phlebovirus was discovered in the Americas, the symptoms of which closely resembled those of SFTS (4). The World Health Organization has since recognized the critical nature of SFTS and listed it among the priority diseases for emergency research and development in 2018 (5).

The pathogen, known as SFTS virus (SFTSV), was initially isolated by the Chinese Center for Disease Control and Prevention (6). The International Committee on Taxonomy of Viruses named the newly discovered virus Dabie bandavirus in 2019, but the designation SFTSV continues to be used worldwide (7). Some of the early-diagnosed SFTS patients had a history of tick bites or found ticks in their living areas before showing symptoms of SFTS, and the sequences amplified from ticks and humans showed a high level of identity, indicating that SFTSV may be transmitted through ticks (8–10). SFTSV can be detected in ticks at different developmental stages and be transmitted by transstadial and transovarial routes (11, 12). SFTSV ribonucleic acid (RNA) has been detected in several tick species, including *Haemaphysalis longicornis* (*H. longicornis*), *Haemaphysalis flava* (*H. flava*), *Ixodes nipponensis* (*I. nipponensis*), and *Amblyomma testudinarium* (*A. testudinarium*), but it can only be isolated from *H. longicornis* ticks (6). Luo et al. (12) found that *H. longicornis* ticks are mainly a reservoir and vector of SFTSV in China. Domestic animals, encompassing goats, cattle, sheep, pigs, dogs, and chickens, are considered potential reservoir hosts for SFTSV. Niu et al. (13) conducted a study focusing on virus isolation and gene sequencing from *H. longicornis* ticks, SFTS patients, and domestic animals at Yantai city in Shandong Province, and it was found that all sequences shared more than 95% identity.

A substantial corpus of studies has filled the gap in the understanding of the transmission of SFTSV between humans and animals and between humans and humans. SFTSV can be detected in the serum, eye swab, saliva, rectal swab, and urine of animals, which underscores the potential for direct zoonotic transmission to humans from animals afflicted with SFTS (14, 15). Nam et al. reported a companion dog that exhibited fever, vomiting, and other SFTS symptoms, which were subsequently confirmed through real-time reverse transcription polymerase chain reaction (PCR) sequencing and an indirect immunofluorescence assay (16). A study from Japan reported that two veterinary personnel were infected with SFTSV from a sick cat, and the whole-genome sequences of the virus isolated from the infected individuals were found to exhibit 100% identity (17). In addition, SFTSV transmission can occur through direct contact of the unprotected skin or mucosa with the bodily fluids of infected humans (18). Exposure to blood or bloody secretions while accompanying patients or while handling a corpse after death is the most common mode of transmission. An observed dose–response relationship between the frequency of contact with blood and the incidence of SFTS infection highlights the elevated risk associated with increased exposure (19). Transmission through aerosols in enclosed environments can also cause SFTSV infections in individuals. Moon et al. (20) investigated the potential nosocomial aerosol transmission of SFTSV and suggested that airborne precautions be adopted to avoid direct contact and droplet transmission. A number of studies reported human-to-human transmission clusters, but only a few quantitatively assessed the human-to-human transmission risk among SFTS clusters.

In this study, we summarized all SFTS cluster outbreaks between 2013 and 2022 in Yantai city, explored the correlations of potential risk factors and SFTSV infection, and quantified the risk factors.

## 2 Methods

### 2.1 Ethical approval and consent statement

This study was reviewed and approved by the Ethics Committee of the Yantai Center for Disease Control and Prevention. It is important to note that our research did not involve any direct interaction with human participants or experimental procedures on humans. The only human materials used were the blood samples collected from SFTS patients and their contacts for public health purposes, and written informed consent for the use of their clinical samples was obtained from these patients.

### 2.2 Key terminology

The diagnosis of confirmed SFTS patients requires three points. First, a person who has a history of working, living, or traveling in hilly, forested, mountainous, or other areas during the epidemic season or has a history of tick bites within 2 weeks of disease onset. Second, a person who develops clinical symptoms such as fever, fatigue, significant anorexia, nausea, and vomiting, accompanied by leukopenia and thrombocytopenia. Third, at least one of the following laboratory findings is required: (1) the identification of SFTSV ribonucleic acid in the patient’s blood samples via molecular diagnostic assays; (2) the successful isolation of the SFTSV; (3) seroconversion or a substantial (> fourfold) increase in the specific IgG antibody titer against SFTSV between acute and recovery serum samples.

SFTS clusters are identified by two or more reported cases of patients with SFTS who work or visit the same locations, such as villages, hills, forests, tea gardens, or scenic areas. In addition, clusters include related cases reported among the close contacts of the initial patient within the longest incubation period of SFTS.

The criteria for identifying human-to-human transmission clusters in SFTS are as follows: (1) Secondary cases of patients who have no history of tick bites, visible tick bite marks on their bodies, or contact with animals in the 2 weeks preceding symptom onset; (2) Secondary cases of patients who have had direct, unprotected contact with the blood, bloody secretions, or other bodily fluids of the primary SFTS case within 2 weeks before their symptom onset; and (3) Secondary cases of patients with the onset of symptoms occurring within the established incubation period following exposure to the primary case.

In our study, the term “index patient” refers to the individual who was first infected with SFTSV through exposure to ticks or other routes.

The secondary attack rate (SAR) refers to the percentage of the affected individuals among all close susceptible contacts between the shortest and longest incubation periods of certain infectious diseases after exposure to a primary case. The calculation formula is as follows:

SAR (%) = number of affected individuals between the shortest and longest incubation periods/total number of susceptible contacts × 100%.

### 2.3 Data source and data collection

The prevention and control of SFTS in China follows national guidelines for notifiable infectious diseases. All suspected and confirmed cases are required to be reported to the National Notifiable Diseases Surveillance System (NNDSS) within 24 h. The clusters are also required to be notified to the National Public Health Emergency Event Surveillance System (PHEESS). The reported data include age, sex, permanent address, occupation, and key dates related to the onset, diagnosis, and outcome. Laboratory-confirmed SFTS cases from 1 January 2013 to 31 December 2022 were retrieved from the NNDSS for analysis.

An epidemiological investigation was carried out by well-trained interviewers using a face-to-face approach. A structured questionnaire was used to collect detailed information about each SFTS cluster. The reacquired information contained demographical information (age, sex, permanent address, occupation, etc.), exposure history within the 1st month before the onset (time, location, cluster settings, and infection route), information about infected individuals and deaths, laboratory test results (IgG titer and whether the SFTSV RNA was positive), and control measures (hospital infection control measures and environmental disinfection).

### 2.4 Data management and analysis

Descriptive epidemiological methods were carried out to analyze the temporal and spatial distribution of the clusters. SARs and an χ^2^ test were used to evaluate the transmissibility and relative risk (RR). A normality test was carried out for between-group comparisons of age and the time interval from the onset to confirmation. In addition, *t*-tests were conducted for the normally distributed data, and χ^2^ tests were conducted for the categorical variables. All calculations were performed using R studio (version 4.3.2; R Foundation for Statistical Computing, Vienna, Austria), and a *p*-value <0.05 was considered to have statistical significance.

## 3 Results

### 3.1 Distribution of the SFTS clusters in China

**Figure 1:**
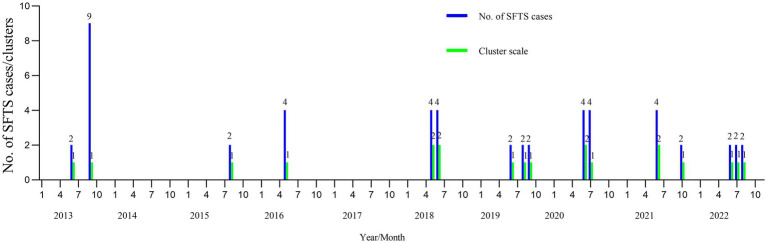
The seasonality of the SFTS clusters in Yantai city from 2013 to 2022. The horizontal axis represents January to December from 2013 to 2012. The vertical axis represents the number of cases and clusters. SFTS, severe fever with thrombocytopenia syndrome.

As shown in Table 1 and Figure 1, 20 SFTS clusters were reported between 2013 and 2022 in Yantai city, Shandong province, with 51 laboratory-confirmed cases, including nine fatalities (17.65%. 9/51). The SFTS clusters showed a seasonal distribution, and the proportions from May to October were 15.69% (8/51), 35.29% (18/51), 11.76% (6/51), 11.76% (6/51), 21.57% (11/51), and 3.92% (2/51), respectively.

**Table 1:** Characteristics of the SFTS clusters in Yantai City, 2013–2022.

Serial code	Time	Location (County)	Cases	Death	Infection route of the index cases	Exposure history / potential human-to-human transmission routes	Human-to-human transmission	Secondary cases (No.)	Places
			(No.)	(No.)					
1	June 2013	Laiyang	2	0	Suspected tick bite	Doing farm work	No	–	Living environment
2	Sep. 2013	Penglai	9	2	Tick bite	Blood and other fluids	Yes	8	Hospital and home
3	Aug. 2015	Haiyang	2	0	Suspected tick bite	Doing farm work	No	–	Living environment
4	May 2016	Qixia	4	1	Suspected tick bite	Doing farm work	Yes	–	Living environment
5	May 2018	Laiyang	2	0	Tick bite	Doing farm work	No	–	Living environment
6	June 2018	Laizhou	2	1	Not known	Doing farm work	No	–	Living environment
7	June 2018	Laizhou	2	0	Not known	Doing farm work	No	–	Living environment
8	May 2018	Penglai	2	0	Tick bite	Doing farm work	No	–	Living environment
9	Sep. 2019	Laishan	2	1	Suspected tick bite	Doing farm work	No	–	Living environment
10	June 2019	Laizhou	2	0	Suspected tick bite	Doing farm work	No	–	Living environment
11	Aug. 2019	Laizhou	2	1	Suspected tick bite	Blood	Yes	1	Hospital
12	June 2020	Qixia	2	0	Not known	Doing farm work	No	–	Living environment
13	July 2020	Zhaoyuan	4	1	Tick bite	Blood and other fluids, aerosols	Yes	3	Hospital and home
14	June 2020	Zhaoyuan	2	0	Suspected tick bite	Doing farm work	No	–	Living environment
15	June 2021	Laizhou	2	0	Suspected tick bite	Doing farm work	No	–	Living environment
16	June 2021	Laizhou	2	1	Tick bite	Doing farm work	No	–	Living environment
17	Oct. 2021	Fushan	2	1	Suspected tick bite	Doing farm work	No	–	Living environment
18	July 2022	Haiyang	2	0	Suspected tick bite	Doing farm work	No	–	Living environment
19	June 2022	Laizhou	2	0	Not known	Doing farm work	No	–	Living environment
20	Aug. 2022	Penglai	2	0	Tick bite	Doing farm work	No	–	Living environment

**Figure 2:**
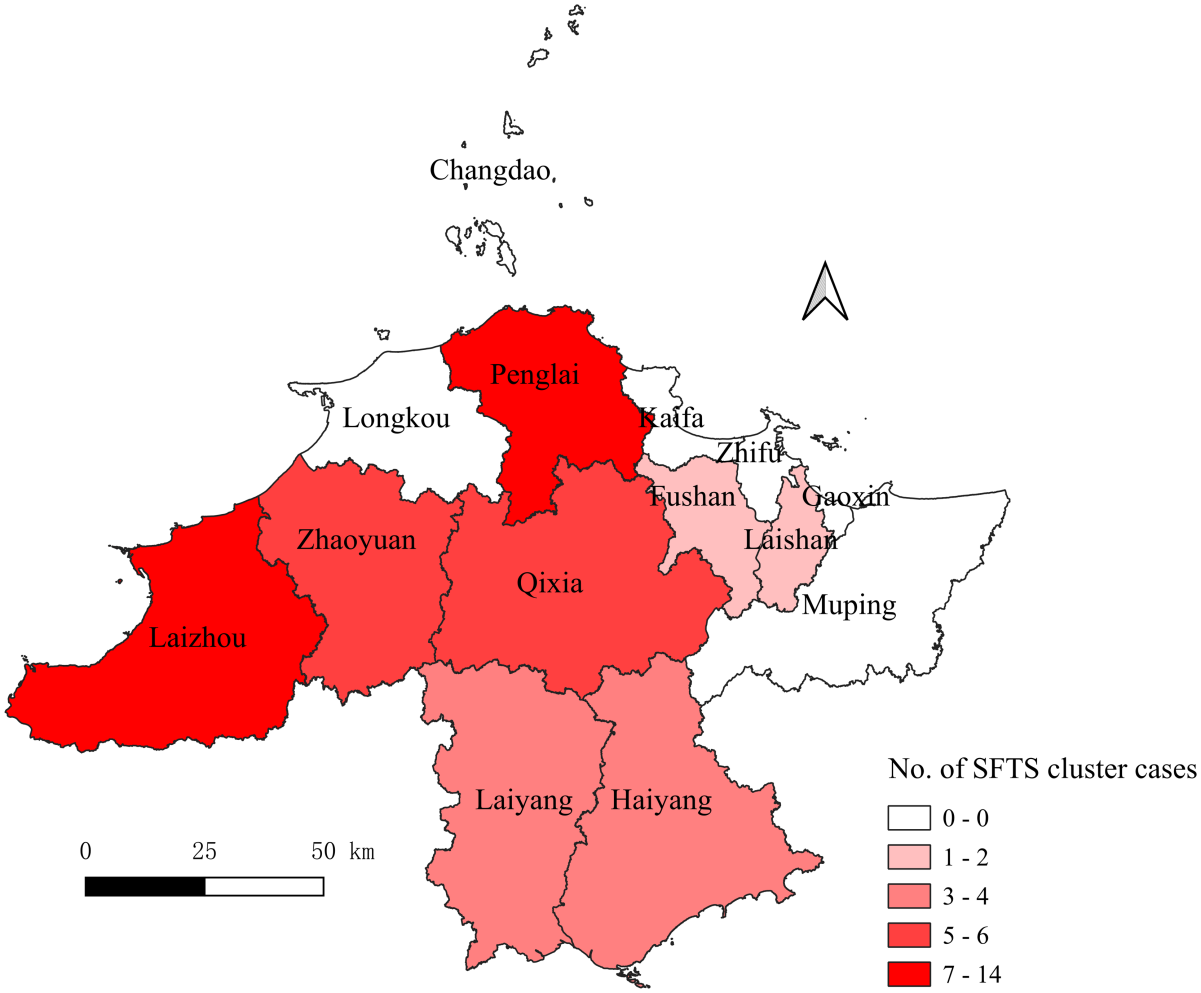
County distributions of the accumulative SFTS cases in Yantai city from 2013 to 2022. SFTS, severe fever with thrombocytopenia syndrome. The numbers indicate the patients from the clusters.

A total of eight counties reported the SFTS clusters. The cases shown in Figure 2 were mainly distributed in Laizhou (*n* = 14), Penglai (*n* = 13), Qixia (*n* = 6), Zhaoyuan (*n* = 6), Haiyang (*n* = 4), Laiyang (*n* = 4), Laishan (*n* = 2), and Fushan (*n* = 2). The distribution of sex was nearly equal, with 26 male patients (50.98%) and 25 female patients (49.02%). The majority of the patients were farmers (74.51%), and 76.47% of the patients were in the 50–79 age group.

### 3.2 Infection routes of the SFTS clusters

**Figure 3:**
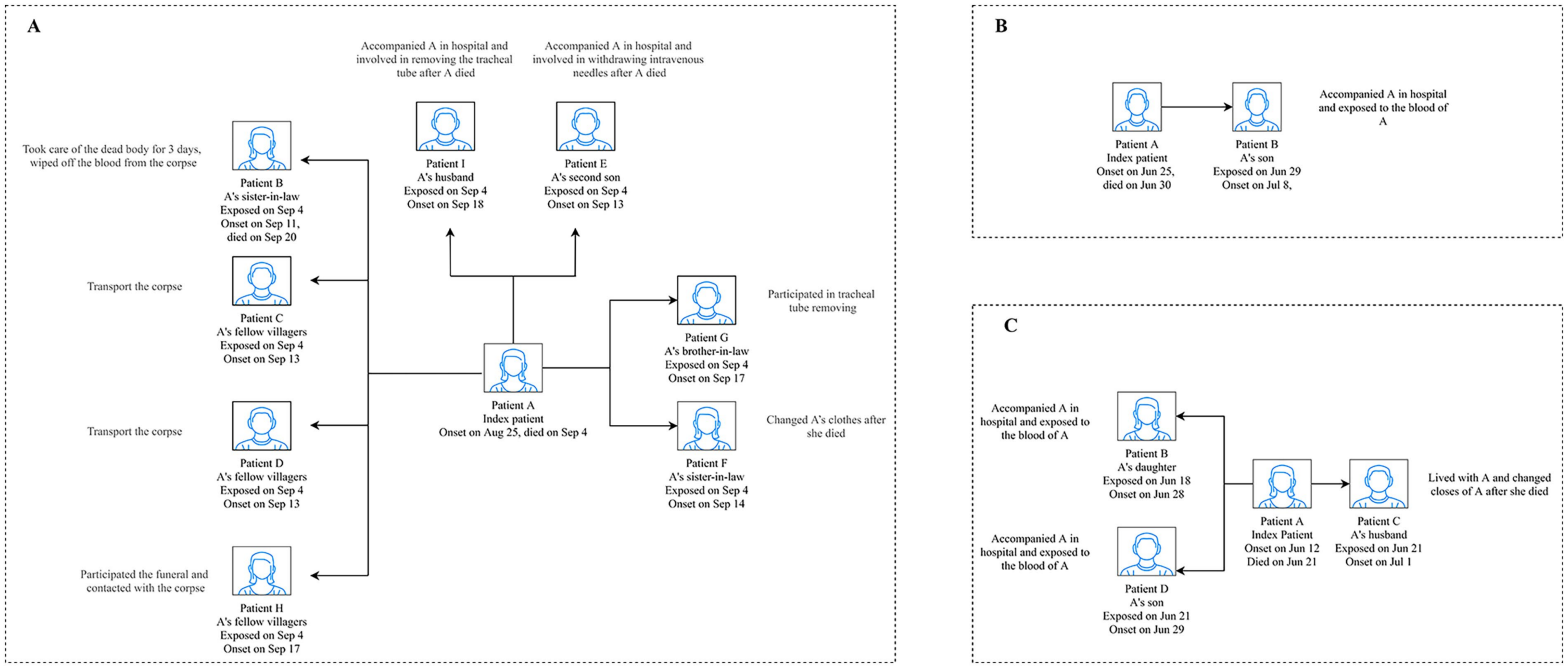
Transmission model of the three human-to-human transmission modes of the SFTS clusters in Yantai city. **A** displays the cluster that occurred in Penglai county in 2013. **B** displays the cluster that occurred in Laizhou county in 2019. **C** displays the cluster that occurred in Zhaoyuan county in 2020. SFTS, severe fever with thrombocytopenia syndrome.

The epidemiological findings revealed that six clusters were due to tick bites, 10 were suspected to be tick-related, and four had unknown infection sources. Of them, three clusters resulted in SFTS secondary cases through human-to-human transmission (one occurred in the hospital and two occurred in both the hospital and the patients’ homes), and the remaining 17 SFTS clusters were caused by the same external environment. Among the three human-to-human clusters, the secondary patients were family members, relatives, and neighbors of the index patients. They were infected with SFTSV through blood contact (i.e., contact with blood or bloody fluids and secretions from the patients) and non-blood contact (i.e., contact with patients’ fluids or secretions other than blood or aerosol). As shown in Figure 3A, Patients I and E took care of the index patient. Along with Patient G, they participated in the removal of medical devices after Patient A died. Other secondary patients came in contact with Patient A’s corpse. Another human-to-human cluster was caused by blood contact of the index patient (Figure 3B). Figure 3C shows that Patients C and D were exposed to the blood of the index patient, while Patient B did not have direct contact with the blood of the index patient. They all remained in a closed space where the patient’s body and her blood-contaminated clothing were stored for a long time. Thus, we speculated that Patients C and D may have been infected with SFTSV through blood or aerosols and Patient B through aerosols.

### 3.3 Risk factors assessment of human-to-human transmission of SFTSV

As shown in Table 2, the SAR of the blood and other fluids contact transmission was higher than that of the non-blood contact transmission [23.33% (7/30) vs. 2.07% (4/193), RR = 14.06, 95% confidence interval (CI) = 3.29–70.65, *p* < 0.001]. In addition, not using personal protection equipment (PPE) showed a higher SAR for the contacts [11.63% (10/86) vs. 2.22% (2/90), RR = 5.74, 95% CI = 1.17–55.44, *p* = 0.013].

**Table 2:** Assessment of the risk factors of the human-to-human transmission of SFTSV.

Transmission route	Exposed population (No.)	Secondary patients (No.)	SAR (%)	χ^2^	*p*	RR (95% CI)
Blood and other fluids contact						
Yes	30	7	23.33	20.70	<0.001	14.06(3.29–70.65)
No	193	4	2.07			
PPE^a^						
No	86	10	11.63	6.12	0.013	5.74(1.17–55.44)
Yes	90	2	2.22			

a PPE, personal protection equipment.

### 3.4 Mortality risk factors among the clusters

Supplementary Table 3 summarizes the risk factors for mortality in the SFTS clusters, which included sex, age, occupation, number of referrals, period from onset to diagnosis, and transmission route. Notably, no significant associations were found between these factors and SFTS-related deaths.

## 4 Discussion

Our study analyzed 20 SFTS clusters in Yantai city (2013–2022), with 17 linked to environmental exposure and 3 to human-to-human transmission. Among the 51 lab-confirmed cases, nine were fatal (17.65%). The clusters were predominantly distributed in Laizhou, Penglai, Qixia, and Zhaoyuan, peaking in summer and autumn. Most patients were farmers aged 50–79 years. Coming in contact with the patients’ blood or other fluids and a lack of PPE significantly correlated with human-to-human SFTSV transmission.

Ticks thrive in mountainous, grassy, or forested areas, with climate factors such as temperature and rainfall influencing their populations, impacting tick-borne viral diseases (21, 22). A study from Brazil showed that female ticks optimally reproduce at 28°C and 80% RH, with lower efficiency and higher mortality at 4°C (23). The peak nymph activity coincides with increased human outdoor activity, elevating exposure risks (24). Deng et al. found that the incidence of SFTS showed an increasing trend as the tick density increased and that the latter was positively correlated with air temperature, wind speed, and sunshine duration (25). Our findings confirmed that SFTS infection peaks in summer and autumn, highlighting the need for heightened tick bite prevention. Yantai city is categorized by mountainous and hilly topography where farmers live and engage in agricultural labor, such as farming, mowing, and herding. These factors increase the risk of exposure to the same natural environment as that of ticks.

All three index patients died, while only one secondary patient died in the SFTS clusters in our study; this finding was similar to that of some studies (26, 27). The clinical symptoms and laboratory findings may explain the secondary patient’s death. Patient B (Figure 3A), along with exhibiting common SFTS symptoms, exhibited skin silt ecchymosis, limb tremor, and respiratory failure and died from disseminated intravascular coagulation. In addition, the patient’s viral load, activated partial thromboplastin time, alanine transaminase (ALT), aspartate aminotransferase (AST), lactate dehydrogenase (LDH), creatine kinase (CK), and creatinine levels were higher than those of the survivors, which may indicate impaired liver, heart, and kidney function. Numerous studies have provided epidemiological evidence of the relationship between these factors and mortality (28–31). The recovery of the other secondary patients could be attributed to more timely diagnosis and treatment, which potentially reduced the viral load compared to the index patients (27).

SFTS patients typically exhibit fever, thrombocytopenia, gastrointestinal symptoms, hemorrhagic tendency, and multiple organ dysfunctions, along with elevated SFTS RNA levels (7). Jeong et al. documented persistently positive SFTSV RNA levels in a patient’s serum samples, along with post-plasma exchange, with positive reactions also observed in the tracheal and gastric aspirates and the urine (S and M segment RNA Ct values ranging from 28.55 to 39.22) (32). In addition, a Japanese study reported SFTSV RNA levels in the cerebrospinal fluid and bronchoalveolar lavage fluid (4.10 and 2.51 log10 copies/mL, respectively), with sputum positivity lasting for roughly 4 months post-onset (33). Zhang et al. collected blood, throat swab, urine, or feces samples from patients with SFTS and found that SFTSV RNA levels can be detected in urine, fecal, and sputum specimens (34). The epidemiological data revealed that most SFTS patients exhibited hemorrhagic symptoms after death (35, 36). In some rural areas of China, when patients are on the brink of death, their families will take them home and after death, hold funerals and then cremate the bodies. The process includes changing clothes, applying makeup, and offering family condolences, and it is highly likely to cause human-to-human transmission of SFTSV. Fang et al. analyzed 27 SFTS clusters from China and Korea and found that direct blood contact, bloody secretion contact, and bloody droplet contact had a higher risk of transmitting SFTSV infection [OR = 6.35 (95% CI: 3.26–12.37), 38.01 (95% CI,19.73–73.23), and 2.27 (95% CI,1.01–5.19)] (26). Similarly, all of our deceased index patients in the SFTS clusters exhibited bleeding, with a significantly higher SAR among those exposed to blood or fluids [23.33% (7/30) vs. 2.07% (4/193)]. Furthermore, contact with blood and other fluids was found to be a significant risk factor for SFTSV transmission (RR = 14.06, 95% CI = 3.29–70.65, *p* < 0.001). This underscores the critical need for caution when handling the bodily fluids of SFTS patients.

The precise mechanisms of blood-to-body SFTSV transmission remain largely unexplored. Potential routes may involve open wounds, compromised skin integrity, and mucosal surfaces of the eyes, mouth, or nose (37). SFTSV disrupts the host’s immunity through a complex cascade, impacting the diverse immune cells, inflammatory mediators, inflammasomes, and signaling pathways (38). Moreover, the compromised adaptive immune response in SFTS patients indicates that the virus induces severe immunological dysregulation by affecting the differentiation of the targeted immune cells (7).

Few secondary patients present open wounds upon exposure to index patients with SFTS, suggesting transmission via oral, nasal, or ocular mucous membranes. The results of a survey on an SFTS cluster indicated that all secondary patients wore gloves and masks during blood contact with the index patient, but none used eye protection (39). Zhou et al. demonstrated that SFTSV could be transmitted through oral or ocular membranes (40). Therefore, it is necessary to wear a full set of PPE when in contact with SFTS patients. In our study, none of the healthcare workers were infected with SFTSV, which may have been due to their standard personal protection. In addition, using PPE during contact could have significantly protected them from SFTSV infection (RR = 5.74, 95% CI = 1.17–55.44, *p* = 0.03).

This study has some limitations, which are worth considering when interpreting its conclusions. First, the data of our study were from the PHEESS, which might not reflect the real-world situation due to the sensitivity of the monitoring system and report bias. However, the information in our study is the best available database regarding SFTS clusters. Second, owing to laboratory constraints, we were unable to obtain viral loads or conduct gene sequence alignments for human-to-human clusters. Therefore, memory biases might have obscured precise exposure histories, although homologous exposures are deemed unlikely. Finally, we did not analyze the risk indicators for the patients who died due to a lack of clinical information.

## 5 Conclusion

In conclusion, we reported 20 SFTS clusters involving 51 laboratory-confirmed patients, which occurred in Yantai city from 2013 to 2022. Contact with patients’ blood or other bodily fluids, along with the absence of PPE, escalates the human-to-human transmission of SFTSV. Thus, health education and behavioral interventions for the relatives and caregivers of SFTS patients, both in hospitals and post-discharge, are crucial.

## Data Availability

The original contributions presented in the study are included in the article/Supplementary material. Further inquiries can be directed to the corresponding author.
