# Supplementary material for: Risk assessment of human-to-human transmission of severe fever with thrombocytopenia syndrome virus based on 10-year clustered analysis
Source: Front Public Health. 2024 Oct 11;12:1419425. doi: 10.3389/fpubh.2024.1419425 (PMC11502313; doi:10.3389/fpubh.2024.1419425)
Supplement: Supplementary file 1 [file Table_1.docx]

**Supplementary table 3 Univariate analysis of risk factors for death in SFTS clusters**

| **Study variables** | **Death** | **Cured patients** | **χ2** | **t** | **P** |
| --- | --- | --- | --- | --- | --- |
|  | 9 | 42 |  |  |  |
| Sex |  |  | 1.362 |  | 0.243 |
| Male | 3(33.33) | 23(54.76) |  |  |  |
| Female | 6(66.67) | 19(45.24) |  |  |  |
| Occupation |  |  | 0.448 |  | 0.503 |
| Farmer | 8(88.89) | 30(71.43) |  |  |  |
| Non-farmer | 1(11.11) | 12(28.57) |  |  |  |
| Age |  |  |  | 1.525 | 0.141 |
| Mean (SD) | 63.89(6.92) | 59.19(13.24) |  |  |  |
| Number of referrals |  |  | 3.981 |  | 0.137 |
| ≤1 | 3(33.33) | 25(62.5) |  |  |  |
| 2 | 5(55.56) | 9(22.5) |  |  |  |
| 3 | 1(11.11) | 6(15) |  |  |  |
| Period from onset to diagnosis (days) | |  |  | 0.203 | 0.842 |
| Mean (SD) | 3.56(1.94) | 3.36(2.12) |  |  |  |
| Transmission route |  |  | 0.434 |  | 0.510 |
| Tick-bite /suspected tick bite | 4(44.44) | 17(40.48) |  |  |  |
| Blood/bodily fluids contact | 1(11.11) | 12(28.57) |  |  |  |
| Not known | 4(44.44) | 13(30.95) |  |  |  |
